# Mental workload during endoscopic sinus surgery is associated with surgeons’ skill levels

**DOI:** 10.3389/fmed.2023.1090743

**Published:** 2023-04-24

**Authors:** Masanobu Suzuki, Kou Miyaji, Kotaro Matoba, Takashige Abe, Yuji Nakamaru, Ryosuke Watanabe, Takayoshi Suzuki, Akira Nakazono, Atsushi Konno, Dominik Hinder, A. J. Psaltis, P. J. Wormald, Akihiro Homma

**Affiliations:** ^1^Department of Otolaryngology-Head and Neck Surgery, Faculty of Medicine and Graduate School of Medicine, Hokkaido University, Sapporo, Hokkaido, Japan; ^2^Graduate School of Information Science and Technology, Hokkaido University, Sapporo, Japan; ^3^Department of Forensic Medicine, Faculty of Medicine and Graduate School of Medicine, Hokkaido University, Sapporo, Hokkaido, Japan; ^4^Department of Urology, Hokkaido University Graduate School of Medicine, Hokkaido University, Sapporo, Hokkaido, Japan; ^5^Department of Surgery–Otorhinolaryngology Head and Neck Surgery, Central Adelaide Local Health Network and the University of Adelaide, Adelaide, SA, Australia

**Keywords:** 3D printer, burnout—professional, endoscopic surgery, surgical training, surgical education, NASA-TLX

## Abstract

**Introduction:**

Surgeons’ mental workload during endoscopic sinus surgery (ESS) has not been fully evaluated. The assessment was challenging due to the great diversity of each patient’s anatomy and the consequence variety of surgical difficulties. In this study, we examined the mental workload of surgeons with various surgical skill levels during ESS under the standardized condition provided by novel-designed 3D sinus models.

**Materials and methods:**

Forty-seven participants performed a high-fidelity ESS simulation with 3D-printed sinus models. Surgeons’ mental workload was assessed with the national aeronautics and space administration-task load index (NASA-TLX). Associations between the total and subscales score of NASA-TLX and surgical skill index, including the board certification status, the number of experienced ESS cases, and the objective structured assessment of technical skills (OSATS), were analyzed. In addition, 10 registrars repeated the simulation surgery, and their NASA-TLX score was compared before and after the repetitive training.

**Results:**

The total NASA-TLX score was significantly associated with OSATS score (*p* = 0.0001). Primary component analysis classified the surgeons’ mental burden into three different categories: (1) the skill-level-dependent factors (temporal demand, effort, and performance), (2) the skill-level-independent factors (mental and physical demand), and (3) frustration. After the repetitive training, the skill-level-dependent factors were alleviated (temporal demand; *z* = −2.3664, *p* = 0.0091, effort; *z* = −2.1704, *p* = 0.0346, and performance; *z* = −2.5992, *p* = 0.0017), the independent factors were increased (mental demand; *z* = −2.5992, *p* = 0.0023 and physical demand; *z* = −2.2509, *p* = 0.0213), and frustration did not change (*p* = 0.3625).

**Conclusion:**

Some of the mental workload during ESS is associated with surgical skill level and alleviated with repetitive training. However, other aspects remain a burden or could worsen even when surgeons have gained surgical experience. Routine assessment of registrars’ mental burdens would be necessary during surgical training to sustain their mental health.

## 1. Introduction

Burnout among medical professionals has been recognized as “a crisis” in modern health care even before Covid-19 (1). A recent nation-wide study in the US demonstrated that 44.0% of physicians had experienced burnout at least once in their career (2). The cost of healthcare related to physician burnout is estimated at between 2.6 to 6.3 billion USD per year (3).

Identified as one of the associated risk factors of burnout in healthcare workers is a high mental workload, along with age, gender, practice setting, specialty, and hours of work per week (2). Mental workload is defined as the total cognitive work needed to accomplish a specific task (4). The National Aeronautics and Space Administration-Task Load Index (NASA-TLX) is designed to evaluate mental workload and widely accepted across many specialties including healthcare (4–7). NASA-TLX allows for evaluation not only of the overall scale of mental workload but also its subscales: mental demand, physical demand, temporal demand, perceived performance, effort, and frustration.

Among various medical procedures, surgery is particularly associated with a high mental and physical burden on surgeon (8–10). It is no wonder that endoscopic sinus surgery (ESS) burdens surgeons mentally because of the risk due to proximity to orbit and brain and requiring fine psychomotor skills (9, 11, 12). So far, however, only few studies focused on surgeons’ mental workload during ESS (9, 11, 12) have been published. One of the reasons is a lack of standardized conditions to evaluate the workload. In general, surgeon’s perceived mental workload is affected by the task’s difficulties (13). The difficulty of ESS largely depends on the complexity of paranasal sinus anatomy, which greatly varies in every patient. Thus, precise assessment of mental workload during ESS has been challenging.

Recently, we reported a high-fidelity ESS simulation surgery using newly designed 3D printed-sinus models, with sufficient face, content, and construct validity (14). With recent advanced 3D-printing technology and the high quality of the printing materials applied, the tactile “real-life” feel of the tissues is reproduced in the models. The mass producibility of the 3D-printed sinus models allows for multiple dissections by surgeons with the exact same anatomy, which is impossible in actual clinical situations. This provides the standardization for comparison among intra- and inter-individuals on several aspects of ESS such as efficiency, efficacy, and safety of surgeries (14, 15).

In this study, we examined surgeons’ mental workload during ESS using NASA-TLX under the standardized condition provided by the 3D-printed sinus models. Specifically, we focused on the possible association between the level of surgical skills and the mental workload. We also examined whether repetitive training could alleviate trainees’ mental workload. This is the first study examining surgeons’ mental workload during ESS under standardized conditions.

## 2. Materials and methods

### 2.1. Participants

This study was conducted concurrently with the previously published studies regarding the validation of 3D-printed sinus models for ESS training (14). Forty-seven otolaryngologists voluntarily took part in the study. Participants were explained the purpose and design of the present study in advance. The written informed consent was obtained from all participants.

### 2.2. Simulation surgeries

For simulation surgeries, a 4-mm rigid nasal endoscope and a monitor (Telepac, Storz, Tuttlingen, Germany), standard ESS instruments (Storz), and a powered microdebrider (Medtronic, Jacksonville, FL) and 3D-printed sinus models (Fusetec, Adelaide, South Australia) were prepared as previously reported (14). The models were 3D-printed from the axial CT scans of actual patients with chronic rhinosinusitis. Infrared reflective markers were attached to several surgical instruments for a motion capture study, although it was not the focus of the present study. The participants were allocated 45 min to complete a unilateral full house ESS (maxillary antrostomy, sphenoethmoidectomy, and frontal sinusotomy). The detail of the simulation surgeries was described in the previous study (14).

All participants performed the surgeries for the 3D-printed sinus models (Model 2 Right side). Further, 10 otolaryngology registrars among the participants repeated the exercise six times as part of repetitive training. In the 1st and the final training (2nd, and 7th surgeries in total, respectively), Model 2 Left was used for intra-individual comparison. Details of the simulation training have been previously reported (14).

### 2.3. Assessment of surgical skill levels

As there is no standard objective method to evaluate the ESS levels, the following index were used in this study; board certification by the Japanese Otolaryngology Society, the number of previous ESS cases performed and the objective structured assessment of technical skills (OSATS) score (16). The scoring system is designed to score surgical performance in each specific procedures during FESS on the 5-likert scale, from the scale of one (unable to perform) to five (performs easily with good flow). A score of three or more in each checklist is considered competent for the task. The OSATS score for ESS was assessed by two attending rhinologists (MS and YN). More details are provided in the previous study (14). Data on the board certification and the number of prior ESS cases were obtained from a questionnaire survey performed after the simulation surgeries. As the definition of ESS experts has not been established, in this study, experts were defined as certified surgeons both within the top 1/3 experienced cases and with top 1/3 OSATS scores. More details of the simulation training and the assessment were described in the previous report (14).

### 2.4. Mental workload assessment

National aeronautics and space administration-task load index (NASA-TLX) were utilized to evaluate mental workload assessment. NASA-TLX is the most widely utilized subjective questionnaire for mental workload assessment and consists of the following six subscales: mental demand, physical demand, temporal demand, perceived performance, effort, and frustration (4–7). After the simulation surgeries, the participants answered the following questions using a 20-point visual analog scale based on the six subscales.

- How much mental activity was required for the surgery? (Mental demand, 1: not at all, 20: extremely high)
- How much physical activity was required for the surgery? (Physical demand, 1: not at all, 20: extremely high)
- How much time pressure did you feel for the surgery? (Temporal demand, 1: not at all, 20: extremely high)
- How successful do you think you were in completing the surgery? (Perceived performance, 1: perfectly completed, 20: nothing achieved at all)
- How hard did you have to work during the surgery? (Effort, 1: not at all, 20: extremely high)
- How insecure, discouraged, irritated, stressed, and annoyed did you feel during the surgery? (Frustration, 1: not at all, 20: extremely high)

### 2.5. Analysis and statistics

Data were shown in median (interquartile range). Shapiro–Wilk tests were applied to evaluate whether the data fitted a normal distribution curve. For analysis on experienced cases, participants were classified into three groups according to the number of experienced cases (the top 1/3, the middle 1/3, and the bottom 1/3 of the experienced cases). As well, they were also classified into three groups according to their OSATS score (the top 1/3, the middle 1/3, and the bottom 1/3 of the score). The total NASA-TLX score were calculated as sum of the 6 subscales. For comparison among three or more groups, data were assessed with the Kruskal-Wallis test followed by the Mann–Whitney U. Principal component analysis (PCA) was performed using the number of experienced cases, OSATS score, and the subscales of NASA-TLX. The paired Wilcoxon test was utilized for comparison before and after the repetitive training. *p* values of less than 0.05 were considered statistically significant. For comparison among three groups or six subscales, the Bonferroni correction was applied and *p* values of less than 0.017 for three groups (i.e., 0.05/3 = 0.017), and 0.0033 for six subscales (i.e., 0.05/15 = 0.0033) were considered statistically significant, respectively. All the analyses were performed using JMP 11 (SAS Institute Inc.).

## 3. Results

### 3.1. Characteristics and the mental workload of the participants

**Figure 1:**
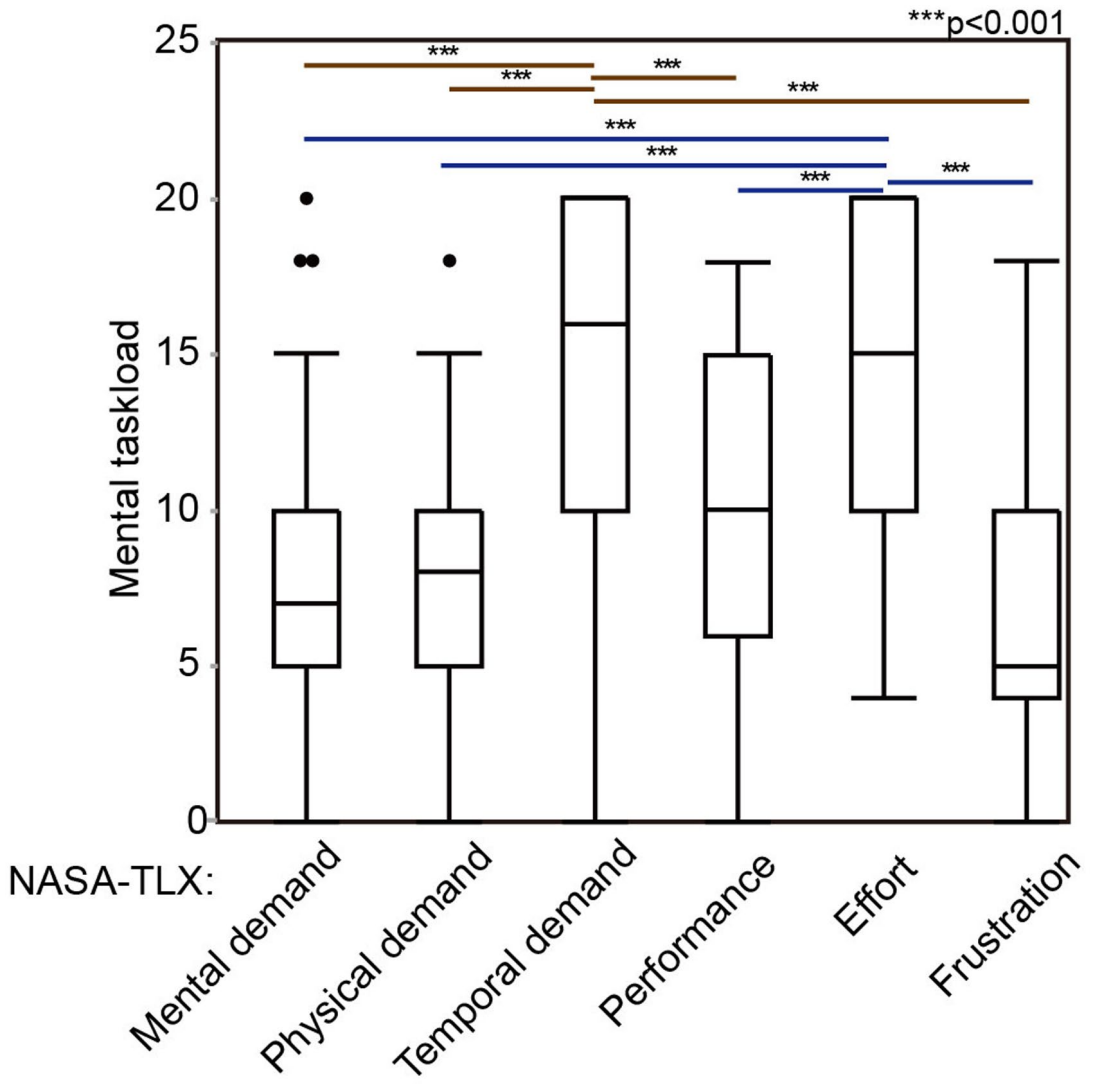
The subscales of mental task load evaluated with NASA-TLX. Forty-seven otolaryngologists performed ESS for 3D-printed sinus models and evaluated their mental workload during ESS, in a point of mental demand, physical demand, performance, effort, and frustration. ESS, endoscopic sinus surgeries; NASA-TLX, national aeronautics and space administration-task load index. ***p < 0.001.

Table 1 shows the characteristics of the 47 participants. Among them, 28 were board-certified members of the Japanese otolaryngology society, and the other 19 were otolaryngology registrars in training. The total NASA-TLX score was 62 (49–75). In the subscales of NASA-TLX, mental demand, physical demand, temporal demand, perceived performance, effort, and frustration were 7 (5–10), 8 (5–10), 16 (10–20), 10 (6–16), 15 (10–20), and 5 (4–10), respectively (Table 1). Temporal demand and effort were significantly higher than the other four subscales (Figure 1).

**Table 1:** Characteristics of participants in the present study.

	All participants (*n* = 47)	Registrars (*n* = 19)	Certified surgeons (*n* = 28)	*p*-value (registrar vs. certified surgeons)	*Z* score (registrar vs. certified surgeons)	Effect size (r; registrar vs. certified surgeons)
OSATS score	55 (44–69)	42 (36–51)	65 (56–75)	**<0.0001**	−4.6200	−1.4610
Work experience in years	10 (3–17)	3 (2–4)	15 (11–21)	**<0.0001**	−5.3410	−1.6890
Surgical experience in number of performed ESS cases	50 (4–100)	2 (0–20)	100 (50–400)	**<0.0001**	−5.3410	−1.6890
NASA-TLX						
Total Score	62 (49–75)	69 (56–81)	52 (39.25–65)	**0.0048**	−2.8410	−0.8984
Mental Demand	7 (5–10)	7 (5–10)	6.5 (4.25–10)	0.8867	−0.1530	−0.0484
Physical Demand	8 (5–10)	8 (5–10)	7.5 (3.5–10)	0.8093	−0.2520	−0.0797
Temporal Demand	16 (10–20)	20 (16–20)	14 (8–19.5)	**0.0054**	−2.7900	−0.8823
Performance (failure = 20)	10 (6–16)	15 (11–16)	7.5 (4.5–10)	**<0.0001**	−4.2320	−1.4610
Effort	15 (10–20)	17 (15–20)	10 (9.25–17.75)	**0.0115**	−2.5390	−0.8029
Frustration	5 (4–10)	5 (4–14)	5.5 (3.25–9.5)	0.6631	−0.4460	−0.1410

NASA-TLX, national aeronautics and space administration-task load index; OSATS, objective structured technical skills assessment.

The bold values means statistically significance.

### 3.2. The surgical skill level was associated with mental workload during ESS

**Figure 2:**
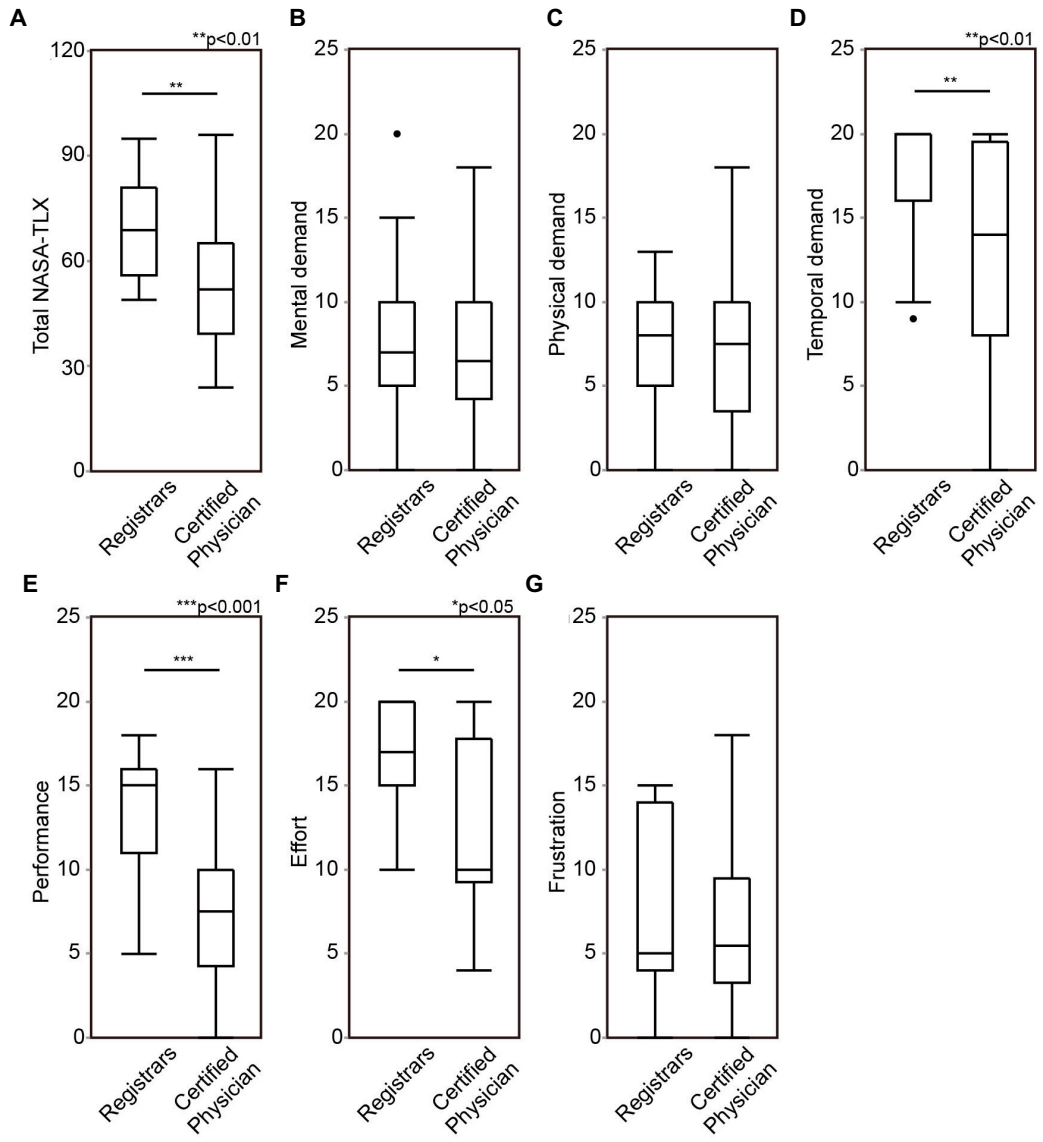
Comparison of mental workload during endoscopic sinus surgery (ESS) between otolaryngology registrars and board-certified otolaryngologists. Total score of national aeronautics and space administration-task load index (NASA-TLX) **(A)**, mental demand **(B)**, physical demand **(C)**, temporal demand **(D)**, performance **(E)**, effort **(F)**, and frustration **(G)** were compared between otolaryngology registrars and board-certified otolaryngologists. ESS, Endoscopic sinus surgeries; NASA-TLX, national aeronautics and space administration-task load index. *p < 0.05, **p < 0.01, ***p < 0.001.

The association between the mental workload during ESS and the surgical skill level was examined. First, the mental workload was compared between the registrars and the certified otolaryngologists. Registrars’ response showed significantly higher total NASA-TLX score (*z* = −2.8410, *p* = 0.0048), higher temporal demand (*z* = −2.7900, *p* = 0.0054), higher performance (*z* = −4.2320, *p* < 0.0001), and higher effort (*z* = −2.5390, *p* = 0.0115) for the simulation surgeries than certified otolaryngologists, while no significant differences were found in mental demand (*z* = −0.1530, *p* = 0.8867), physical demand (*z* = −0.2520, *p* = 0.8093), and frustration (*z* = −0.4460, *p* = 0.6631, Figure 2 and Table 1).

Next, the mental workload was analyzed in association with the number of the experienced cases. The bottom 1/3 of the cases showed significantly, higher total NASA-TLX score (*z* = −3.3623, *p* = 0.0008), higher temporal demand (*z* = −4.3109, *p* < 0.0001), higher performance (*z* = −4.1756, *p* = 0.0026), and higher effort (*z* = −3.7960, *p* = 0.0006) than the top 1/3, while there was no significant difference in mental demand (*z* = 3.2176, *p* = 0.7179), physical demand (*z* = 0.2799, *p* = 0.7795), and frustration (*z* = −1.7878, *p* = 0.2406), between the top 1/3, middle 1/3, and bottom 1/3 of the surgeons who were experienced (Supplementary Figure 1) and Table 1.

For the OSATS score, the bottom 1/3 had a significantly higher total NASA-TLX score (*z* = −3.835, *p* = 0.0001), higher temporal demand (*z* = −3.7034, *p* = 0.0002), higher performance (*z* = −4.4203, *p* = 0.0016), and higher effort (*z* = −3.6567, *p* = 0.0002) than the top 1/3 (Supplementary Figure 2 and Table 2). There were no significant differences in physical demand (*z* = 0.0599, *p* = 0.9895), and frustration (*z* = −1.9276, *p* = 0.0837) among the groups. In mental demand, although the Kruskal Wallis test showed a low value of *p* among the groups (*p* = 0.0360), the comparison between the groups did not reach statistical significance.

**Figure 3:**
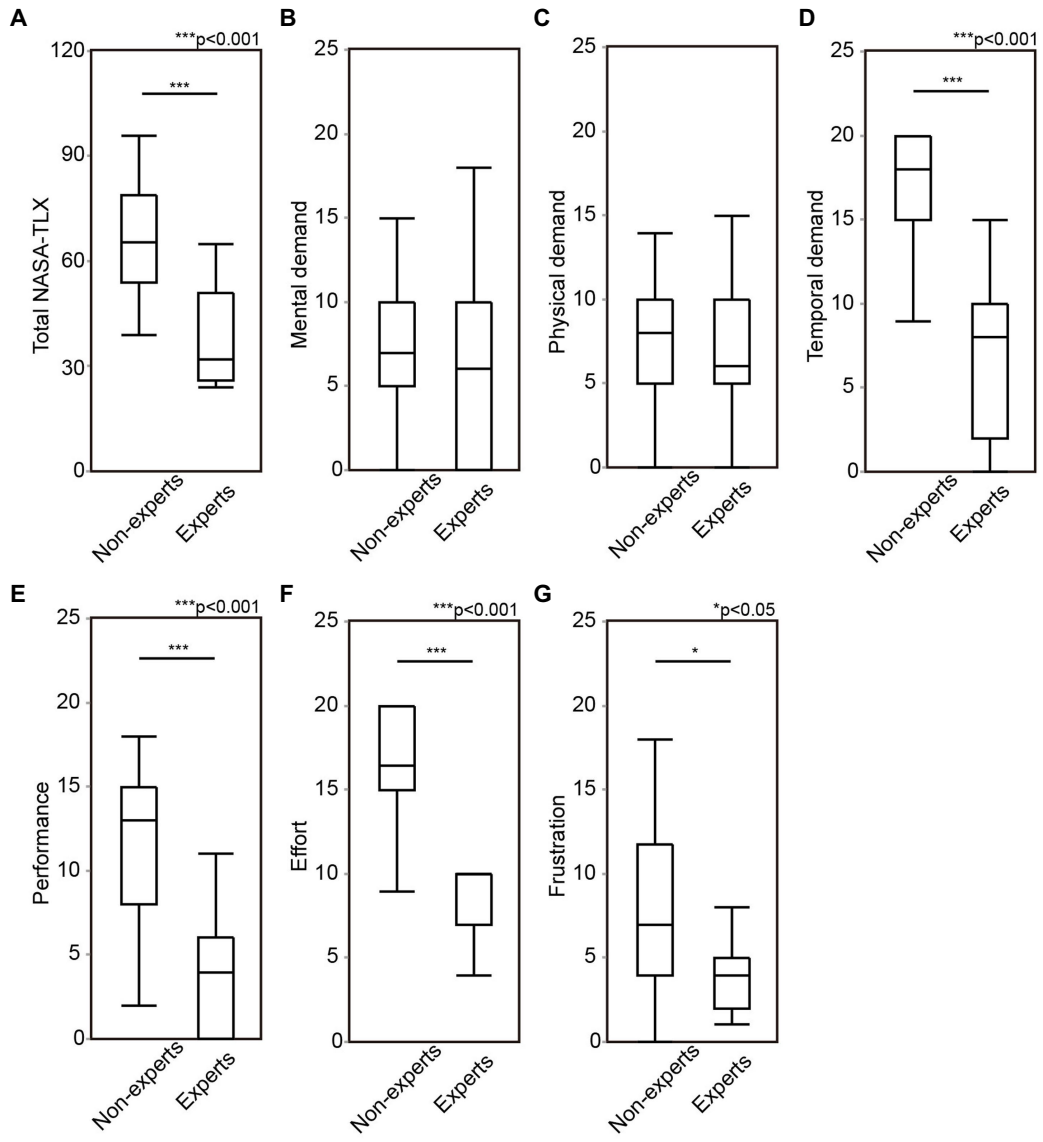
The comparison of mental workload during ESS between experts and non-experts. Total score of NASA-TLX **(A)**, mental demand **(B)**, physical demand **(C)**, temporal demand **(D)**, performance **(E)**, effort **(F)**, and frustration **(G)** were compared between experts and non-experts. ESS, endoscopic sinus surgeries; OSATS, objective structured assessment of technical skills; NASA-TLX, national aeronautics and space administration-task load index. *p < 0.05, **p < 0.01, ***p < 0.001.

The mental workload was compared between the experts and non-experts. The experts showed significantly lower score in total NASA-TLX score (*z* = −4.1354, *p* < 0.0001), temporal demand (*z* = −4.48429, *p* < 0.0001), performance (*z* = −4.0808, *p* < 0.0001), effort (*z* = −3.9414, *p* < 0.0001), and frustration (*z* = −1.9941, *p* = 0.0461), compared to non-experts (Figure 3 and Supplementary Table 3). These results suggest that surgeons’ mental workload during ESS is associated with surgical skill level, similar to what has been previously reported in other surgical disciplines (17). This was especially true for temporal demand, performance and effort.

**Figure 4:**
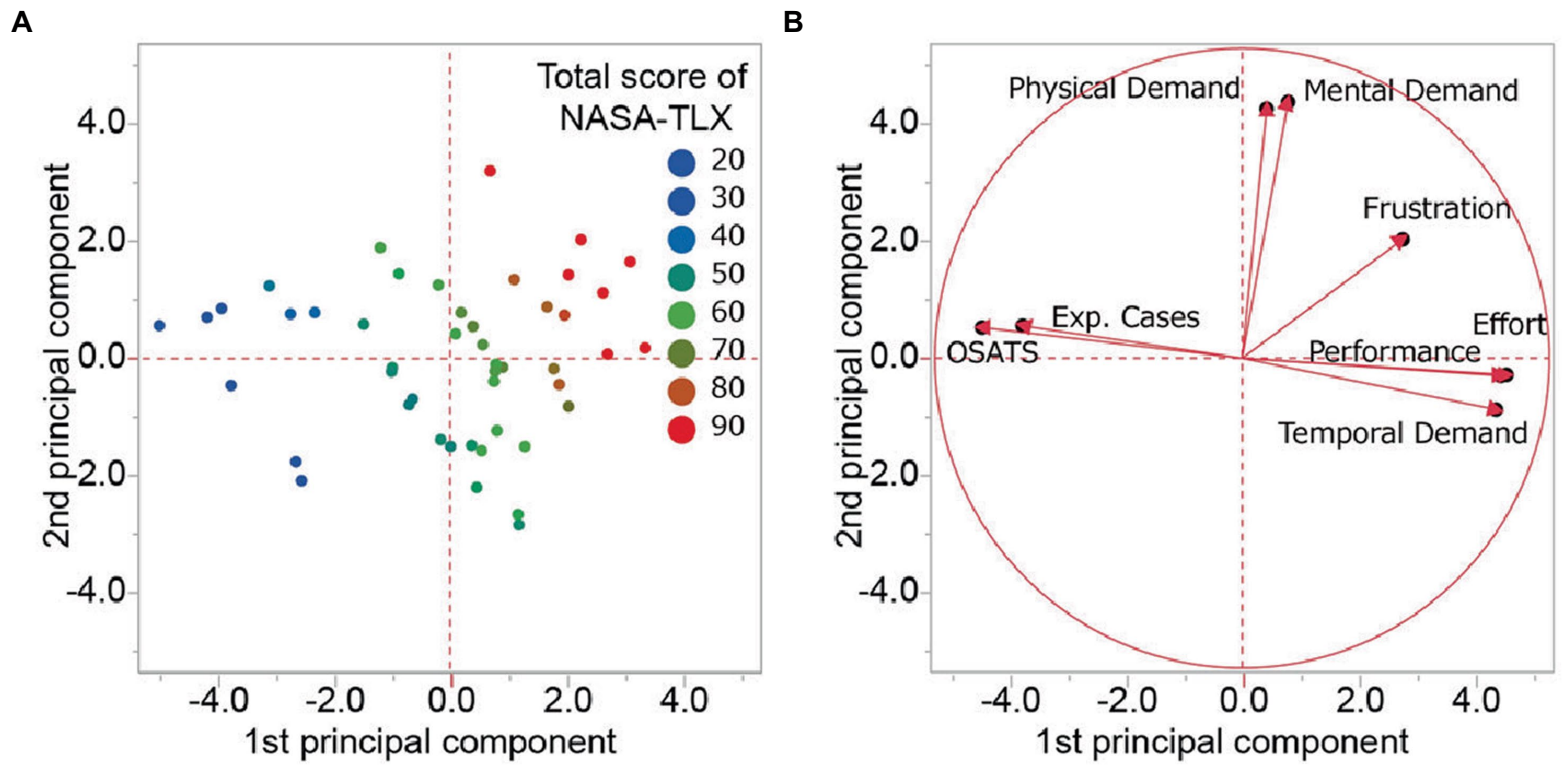
Principal component analysis regarding the surgical skill level and mental workload. **(A)** The principal component score plot on each surgeon. The color represents the total score of NASA-TLX (red; high, green; middle, and blue low). **(B)** The Loading plots of 1st and 2nd principal components. NASA-TLX, national aeronautics and space administration-task load index; Exp. Cases, experienced cases; OSATS, objective structured assessment of technical skills.

Next, the index related to the surgical skill level (the number of previous surgical cases and OSATS score) and the subscales in NASA-TLX were subjected to PCA test (Figure 4 and Table 2). PCA demonstrated that surgeons with low mental workload were mainly distributed to the left side of the principal component score plot and those who had a middle and high mental workload to the center and the right of the plot. The 1st, 2nd, and 3rd principal components explained 45.8, 19.2, and 10.6% of the total variance. The 1st principal component was the most strongly affected by temporal demand (loading 0.86), followed by OSATS score (−0.85), effort (0.84), performance (0.83) and the number of prior ESS cases (−0.71). The three subscales in the 1^st^ component, temporal demand, effort, and performance, were negatively correlated to the index, prior surgical cases and OSATS score (Table 2). The 2nd principal component consisted of mental demand (loading 0.83) and physical demand (0.81). The two subscales are independent from both the number of cases and OSATS score (Table 2). The 3rd principal component was affected by frustration (loading 0.64). This implies that the mental workload that the surgeon felt during ESS consisted of a different type of burden with a part of the burden associated with the surgical skill level other parts were independent of the surgeons’ experience.

**Table 2:** Correlation coefficient among the surgical skill level of ESS and the subscales of NASA-TLX.

	Indexes for surgical skill level		NASA-TLX					
	Exp. Cases	OSATS	Mental demand	Physical demand	Temporal demand	Performance	Effort	Frustration
Exp. cases	-	**0.48**	−0.08	−0.01	**−0.67**	**−0.45**	**−0.55**	−0.15
OSATS	**0.48**	-	−0.05	−0.00	**−0.63**	**−0.76**	**−0.62**	−0.39
Mental demand	−0.08	−0.05	-	**0.44**	0.09	−0.02	0.07	0.27
Physical demand	−0.01	−0.00	**0.44**	-	0.05	−0.03	0.04	0.17
Temporal demand	**−0.67**	**−0.63**	0.09	0.05	-	**0.61**	**0.70**	0.34
Performance	**−0.45**	**−0.76**	−0.02	−0.03	**0.61**	-	**0.63**	0.36
Effort	**−0.55**	**−0.62**	0.07	0.04	**0.70**	**0.63**	-	0.37
Frustration	−0.15	−0.39	0.27	0.17	0.34	0.36	0.37	-

A correlation coefficient <−0.4 or >0.4 were shown in bold. NASA-TLX, national aeronautics and space administration-task load index; Exp. cases, Experienced ESS cases; OSATS, objective structured technical skills assessment.

### 3.3. Changes noted in the registrars’ mental workload after repetitive ESS training

**Figure 5:**
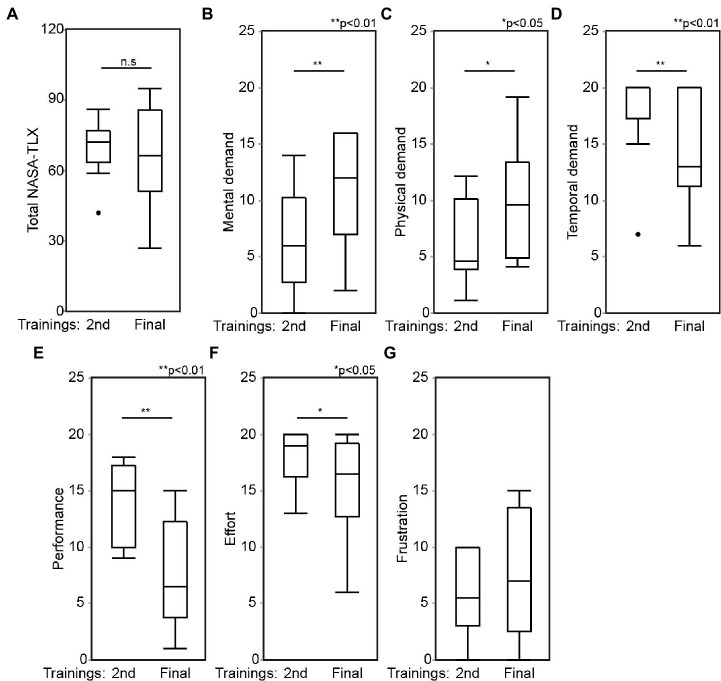
Registrars’ mental workload during ESS before and after the repetitive training. After the first simulation surgeries, 10 registrars repeated surgeries more six times. Total score of NASA-TLX **(A)**, mental demand **(B)**, physical demand **(C)**, temporal demand **(D)**, performance **(E)**, effort **(F)**, and frustration **(G)** were compared between 2nd training and the final training, which the same models were used. ESS, Endoscopic sinus surgeries; NASA-TLX, national aeronautics and space administration-task load index. 2nd, the 2nd training; Final, the final training.

Ten registrars among the participants performed mock surgeries an additional six times as part of a repetitive training program. Their skill improvement was investigated in detail and confirmed in the previous study (14). Briefly, their OSATS score significantly increased after the program (*z* = −2.8031, *p* < 0.001). The residual bony septation within paranasal sinuses, evaluated with CT examination, was also significantly decreased (*z* = −3.326, *p* = 0.013) (14). The NASA-TLX was compared in the 2nd and final training, where the same 3D model was used. Although there were no significant differences in the total NASA-TLX, most subscales significantly changed. Mental and Physical demand significantly increased after the repetitive training, while temporal demand, performance, and effort were significantly decreased (Figure 5 and Supplementary Table 4). There was no significant change in frustration after the training.

## 4. Discussion

So far, mental workload during ESS has not been able to be fully evaluated due to the significant variation in anatomy between patients. The first paper reported by Alobid, et al., studied 15 novice surgeons performing ESS on actual patients and evaluated their mental workload including the surgeons’ anxiety score, cardiovascular index, and serum cortisol level (9). They found that the surgeons had a high anxiety score with increased blood pressure and cortisol levels during ESS (11). Stelter et al. investigated four experienced rhinologists’ mental workload during transnasal endoscopic surgeries for variety of diseases including chronic rhinosinusitis, mucoceles of frontal sinuses, cerebral spinal fluid leaks, and skull-base surgeries (12). Mental workload also has been investigated as part of studies on utility of new devices, such as augmented reality (AR) image guidance (18), Virtual reality (VR) (19), and flexible endoscopes for ESS (20). In addition, the role of surgical ergonomics on the surgeons’ mental workload was evaluated (21, 22). However, the major limitation common to all these studies was the limited number of participants and the unstandardized conditions due to the great diversity of paranasal sinus’ anatomies in the patients or cadaveric materials on which the surgery was performed.

This study was performed with 47 participants under the standardized conditions provided by the 3D sinus model. This allows for a more detailed analysis on mental workload during ESS. We found that total mental workload was significantly associated with surgeons’ skill level. As for the subscales, temporal demand, effort, and performance, these were significantly associated with surgical experience (number of prior surgeries), while the mental and physical demand had no relationship with experience. PCA demonstrated that the surgeons’ mental burden during ESS could be classified into three categories: (1) the surgical experience-dependent factor (temporal demand, effort, and performance), (2) the surgical experience-independent factor (mental and physical demand), and (3) the in-between factor (frustration). Interestingly, these three categories significantly changed after repetitive training; (1) the experience dependent factors were alleviated, (2) the mental and physical factors improved, and (3) the in-between factor (frustration) did not change. As a result, the total NASA-TLX was unchanged after the training. However, training improved the most burdensome factors of temporal demand and effort. Interestingly, the mental and physical demand was increased after the repetitive training. This suggests that some mental burdens are not always alleviated after training but remain similar or even worsen even when surgeons gain surgical experience. A higher physical demand was found in surgeons with previous burnout experience (*p* = 0.048, data not shown). Evaluation of surgical skills and the routine assessment of mental burden should be part of surgical training to allow the continued well being of trainees’ mental health.

Surgical residents are continuously exposed to a significant amount of mental burden (23, 24). The assessment of mental workload should be considered when residency programs are designed (23) and appropriate adjustments made. A high mental workload in surgery increases the risk of complications (25, 26). As the development of residency programs continue to evolve so the mental stress of the program should form a significant part of improving the program and consequently the health of the residents. Therefore, it is important that there is a standardized method of assessing mental health.

The significant improvement in the ability of 3D printers to generate models from actual patients for residents to perform surgery in temporal bones (27–29), paranasal sinuses (30–33), skull base (30, 34–37), kidney, renal pelvis, ureter (38, 39), spine (40, 41), mandibula (30), aorta (42) and heart (43) has been established. The 3D-printed models are not only useful for surgical training (44), but also provide a platform to assess surgeons’ mental workload during surgery (39–41). With this improved technology there is now a high degree of similarity and reality for surgeons as has been shown in recent studies (39). The 3D-printed sinus models used in this study have been previously studied and have shown satisfactory face, content, and construct validity (14). There is a significantly high correlation between the assessment of the 3D-printed sinus models when compared with cadaveric materials both in terms of skill levels and surgical efficiency (skill levels; r = 0.828 and efficiency; r = 0.953) (14). The high fidelity of the 3D-printed sinus models also allows for the prediction of mental workload in ESS for actual patients in advance. The other significant advantage of the 3D-printed sinus models is that they are plastic and do not any ethical and transport restrictions, unlike cadaveric materials. This is illustrated by a recent published study for a remote surgical training course held simultaneously in Japan and Australia with combination of the 3D models and a web conferencing systems (15). This provides a standardized surgical exercise on complex anatomy that can be used to evaluate surgeons’ performance, skill level and can include mental workload. There is now the ability with the number of different anatomies available for surgical boards to be able to use these standardized yet complex anatomies to evaluate actual surgical skill rather than just the theory of surgery.

This study has limitations because although simulation training using the 3D sinus model has been shown to have a high fidelity and has been previously validated (14), there are still small differences between the models and actual ESS on patients. The models reduce the fear of intraoperative bleeding and the risk of operative complications, and this can affect the surgeon’s mental workload. In addition, in this study all surgeons were asked to perform the surgery within a specified time and this may add to the temporal demand. It has been previously shown that time pressure is the biggest stressor for surgeons during ESS (12). Some differences only had small effect sizes despite their statistically significant (*cf.* a difference in frustration between experts and non-experts, Supplementary Table 3). However, most other statistically significant differences analyzed in this study also held sufficient effect sizes. The total NASA-TLX score in this study was not a weighted score but just a summation of raw figures of the subscales, although the raw total score was validated as sensitive as the weighted score (45). It is also to be investigated if the changes in the mental workload found after the short-term training are equal to the differences in the workload found among the surgeons with various skill levels produced by their long-term experience. Further, our study lacks the objective method to evaluate the workload such as heart rate, blood pressure, catecholamines or cortisol in blood and saliva (46). Despite the limitations, this study is the first step to assess the mental workload during ESS under the standardized conditions and can help with the development of surgical training curricula in the future.

## 5. Conclusion

The level of surgical skill significantly affected surgeons’ mental workload during ESS, especially in temporal demand, performance, and effort.

## Data availability statement

The raw data supporting the conclusions of this article will be made available by the authors, without undue reservation.

## Ethics statement

Ethical review and approval was not required for the study on human participants in accordance with the local legislation and institutional requirements. The patients/participants provided their written informed consent to participate in this study.

## Author contributions

MS, TA, AK, and AH designed the project. MS, KMi, RW, TS, AN, YN, and AH organized the simulation training and collected data. MS and YN analyzed OSATS score. KMa, and AK analyzed NASA-TLX score. MS, DH, AP, and PW wrote the draft. All authors contributed to the article and approved the submitted version.

## Funding

This work was partially supported by JSPS Grant-in-Aid for Scientific Research JP18H04102, 22 K16923, and 22 K10599.

## Conflict of interest

PW: consultant for Fusetec and receiving royalties from Fusetec. AP: consultant for Fusetec, Medtronic, ENT technologies, Tissium, and Aerin Medical, shareholder of Chitogel, and speaker’s bureau for Sequiris.

The remaining authors declare that the research was conducted in the absence of any commercial or financial relationships that could be construed as a potential conflict of interest.

## Publisher’s note

All claims expressed in this article are solely those of the authors and do not necessarily represent those of their affiliated organizations, or those of the publisher, the editors and the reviewers. Any product that may be evaluated in this article, or claim that may be made by its manufacturer, is not guaranteed or endorsed by the publisher.

## Supplementary material

SUPPLEMENTARY FIGURE S1 The comparison of mental workload during ESS in association with surgeons’ experienced ESS cases. Participants were classified into three groups according to the number of prior surgeries performed (the top 1/3, the middle 1/3, and the bottom 1/3). Total score of NASA-TLX **(A)**, mental demand **(B)**, physical demand **(C)**, temporal demand **(D)**, performance **(E)**, effort **(F)**, and frustration **(G)** were compared among the three groups. ESS, Endoscopic sinus surgeries; NASA-TLX, National Aeronautics and Space Administration-Task Load Index. ***p* < 0.01, ****p* < 0.001.

SUPPLEMENTARY FIGURE S2 The comparison of mental workload during ESS in association with OSATS score. Participants were classified into three groups according to their OSATS score (the top 1/3, the middle 1/3, and the bottom 1/3). Total score of NASA-TLX **(A)**, mental demand **(B)**, physical demand **(C)**, temporal demand **(D)**, performance **(E)**, effort **(F)**, and frustration **(G)** were compared amongst these three groups. ESS, Endoscopic sinus surgeries; OSATS, Objective Structured Assessment of Technical Skills; NASA-TLX, National Aeronautics and Space Administration-Task Load Index. ***p* < 0.01, ****p* < 0.001.
