# Supplementary figures and images for: Mental workload during endoscopic sinus surgery is associated with surgeons’ skill levels
Source: Front Med (Lausanne). 2023 Apr 24;10:1090743. doi: 10.3389/fmed.2023.1090743 (PMC10165102; doi:10.3389/fmed.2023.1090743)

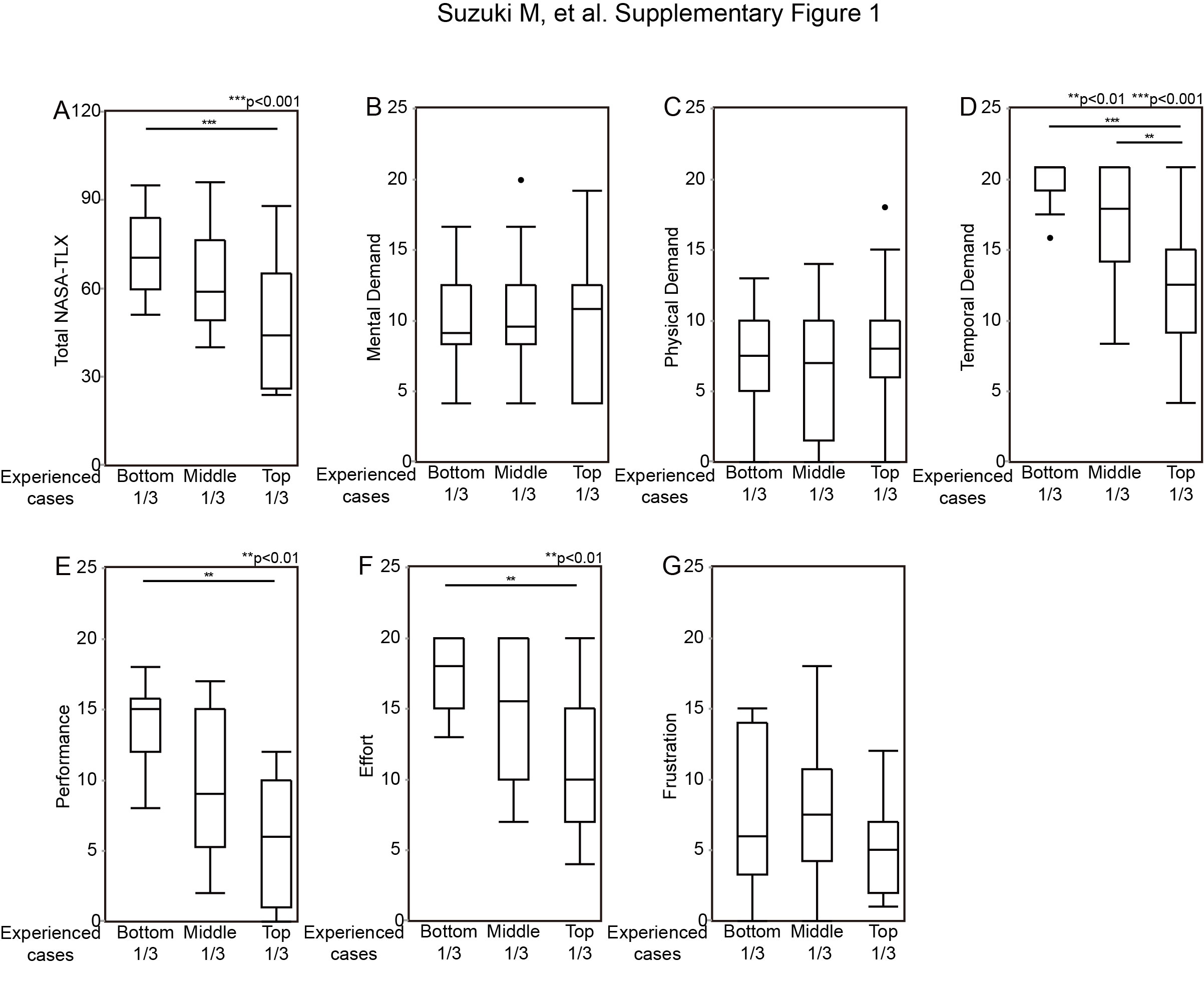

Supplement: SUPPLEMENTARY FIGURE S1 — The comparison of mental workload during ESS in association with surgeons’ experienced ESS cases. Participants were classified into three groups according to the number of prior surgeries performed (the top 1/3, the middle 1/3, and the bottom 1/3). Total score of NASA-TLX (A), mental demand (B), physical demand (C), temporal demand (D), performance (E), effort (F), and frustration (G) were compared among the three groups. ESS, Endoscopic sinus surgeries; NASA-TLX, National Aeronautics and Space Administration-Task Load Index. **p < 0.01, ***p < 0.001. [file Image_1.JPEG]

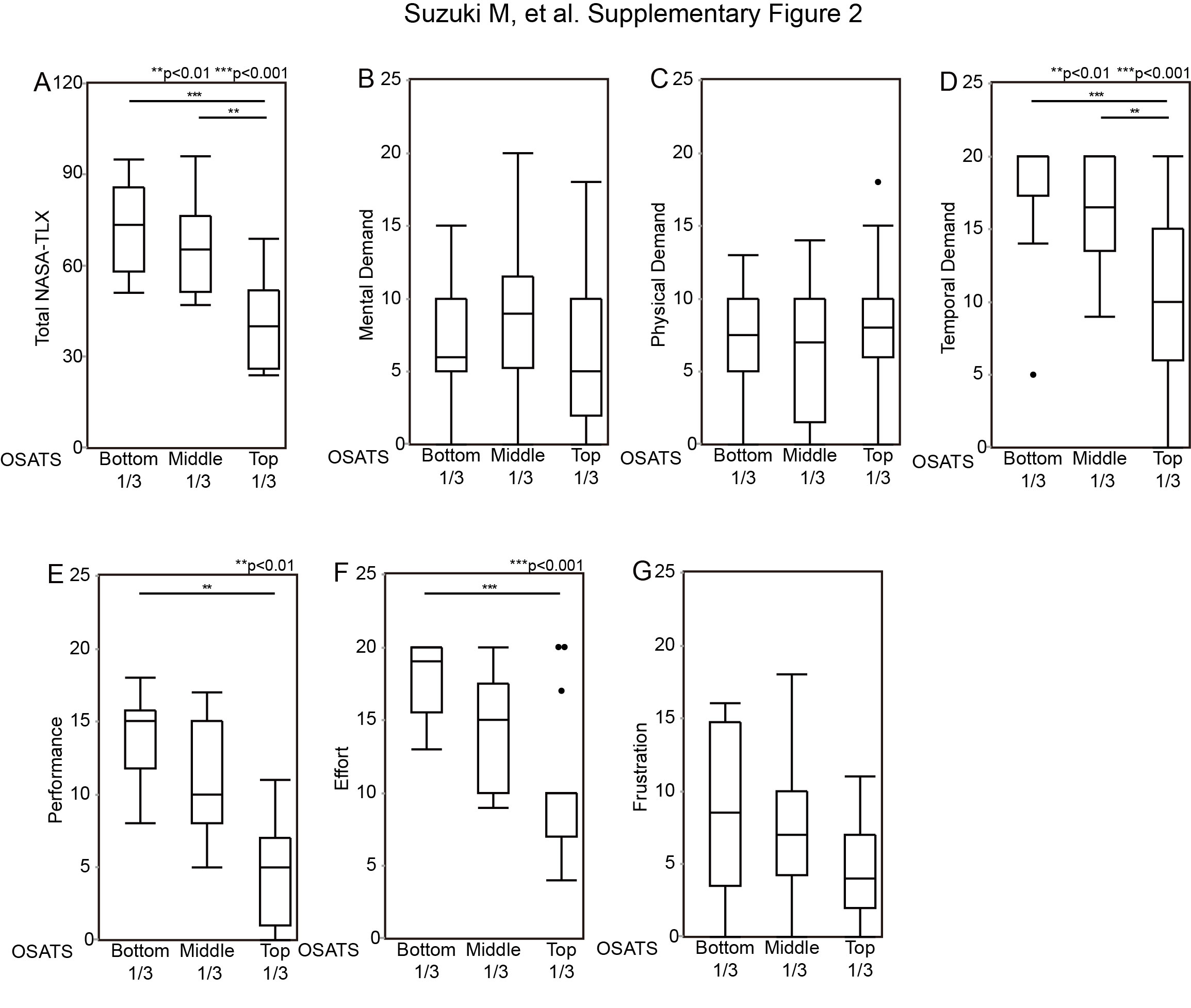

Supplement: SUPPLEMENTARY FIGURE S2 — The comparison of mental workload during ESS in association with OSATS score. Participants were classified into three groups according to their OSATS score (the top 1/3, the middle 1/3, and the bottom 1/3). Total score of NASA-TLX (A), mental demand (B), physical demand (C), temporal demand (D), performance (E), effort (F), and frustration (G) were compared amongst these three groups. ESS, Endoscopic sinus surgeries; OSATS, Objective Structured Assessment of Technical Skills; NASA-TLX, National Aeronautics and Space Administration-Task Load Index. **p < 0.01, ***p < 0.001. [file Image_2.JPEG]
