# Supplementary material for: Mental workload during endoscopic sinus surgery is associated with surgeons’ skill levels
Source: Front Med (Lausanne). 2023 Apr 24;10:1090743. doi: 10.3389/fmed.2023.1090743 (PMC10165102; doi:10.3389/fmed.2023.1090743)
Supplement: Supplementary file 3 [file Table_1.DOCX]

Supplementary Table 1. Mental workload among the three groups classified with the number of experienced ESS cases.

|  | **Experienced ESS cases** | | | **P value** | | | | | | | **Z score** | | | **Effect size (r)** | | |
| --- | --- | --- | --- | --- | --- | --- | --- | --- | --- | --- | --- | --- | --- | --- | --- | --- |
|  | **Bottom 1/3**  **(n=16)** | **Middle 1/3**  **(n=16)** | **Top 1/3**  **(n=15)** | **Kruskal Wallis** | **Bottom vs. Middle** | | **Middle vs. Top** | | **Bottom vs. Top** | | **Bottom vs. Middle** | **Middle vs. Top** | **Bottom vs. Top** | **Bottom vs. Middle** | **Middle vs. Top** | **Bottom vs. Top** |
| Surgical experience in number of performed ESS cases | 1.5 (0-4.75) | 50 (30-50) | 400 (100-1000) | **<.0001** | | **<.0001** | | **<.0001** | | **<.0001** | 4.8459 | 4.5870 | 4.7651 | 0.7068 | 0.6691 | 0.6951 |
| OSATS score | 38 (35.25-45) | 55.5 (51-64.5) | 73 (63-77) | **<.0001** | | **0.0003** | | **<.0001** | | **<.0001** | 4.2824 | 3.6612 | 4.7289 | 0.6247 | 0.5340 | 0.6898 |
|  |  |  |  |  | |  | |  | |  |  |  |  |  |  |  |
| NASA-TLX |  |  |  |  | |  | |  | |  |  |  |  |  |  |  |
| Total Score | 70.5 (59.75-84) | 59 (49.25-76.5) | 44 (26-65) | **0.0013** | | 0.0703 | | 0.0242 | | **0.0008** | -1.8100 | -2.2545 | -3.3623 | -0.2640 | -0.3289 | -0.4904 |
| Mental Demand | 6 (5-10) | 6.5 (5-10) | 8 (0-10) | 0.8871 | | 0.6608 | | 1 | | 0.7179 | 3.2756 | 3.2382 | 3.2176 | 0.4778 | 0.4723 | 0.4693 |
| Physical Demand | 7.5 (5-10) | 7 (1.5-10) | 8 (6-10) | 0.6647 | | 0.5674 | | 0.3889 | | 0.7795 | -0.5718 | 0.8617 | 0.2799 | -0.0834 | 0.1257 | 0.0408 |
| Temporal Demand | 20 (18-20) | 16.5 (12-20) | 10 (6-13) | **<.0001** | | 0.0436 | | **0.003** | | **<.0001** | -2.0175 | -2.9701 | -4.3109 | -0.2943 | -0.4332 | -0.6288 |
| Performance  (failure=20) | 5 (4.25-8) | 11 (5-14.75) | 13 (9-18) | **0.0043** | | 0.0128 | | 0.3611 | | **0.0026** | -2.4893 | -2.0850 | -4.1756 | -0.3631 | -0.3041 | -0.6091 |
| Effort | 18 (15-20) | 15.5 (10-20) | 10 (7-15) | **0.0006** | | 0.1291 | | 0.0224 | | **0.0001** | -1.5175 | -2.2834 | -3.7960 | -0.2214 | -0.3331 | -0.5537 |
| Frustration | 6 (3.25-14) | 7.5 (4.25-10.75) | 5 (2-7) | 0.2088 | | 0.8202 | | 0.0738 | | 0.2406 | 0.2273 | -1.1735 | -1.7878 | 0.0332 | -0.1712 | -0.2608 |

NASA-TLX: National Aeronautics and Space Administration-Task Load Index. OSATS: objective structured technical skills assessment.

Supplementary Table 2. Mental workload among the three groups classified with OSATS score.

|  | **OSATS score** | | | **P value** | | | | | | | **Z score** | | | **Effect size (r)** | | |
| --- | --- | --- | --- | --- | --- | --- | --- | --- | --- | --- | --- | --- | --- | --- | --- | --- |
|  | **Bottom 1/3**  **(n=16)** | **Middle 1/3**  **(n=16)** | **Top 1/3**  **(n=15)** | **Kruskal Wallis** | **Bottom vs. Middle** | | **Middle vs. Top** | | **Bottom vs. Top** | | **Bottom vs. Middle** | **Middle vs. Top** | **Bottom vs. Top** | **Bottom vs. Middle** | **Middle vs. Top** | **Bottom vs. Top** |
| OSATS score | 38 (35.25-44.75) | 55.5 (51.5-58.75) | 73 (69-77) | **<.0001** | | **<.0001** | | **<.0001** | | **<.0001** | 4.8106 | 4.7299 | 4.7299 | 0.7017 | 0.6899 | 0.6899 |
| Surgical experience in number of performed ESS cases | 1.5 (0-8.75) | 50 (30-100) | 400 (50-1000) | **<.0001** | | **<.0001** | | **0.002** | | **<.0001** | 3.9202 | 3.0955 | 4.6398 | 0.5718 | 0.4515 | 0.6768 |
|  |  |  |  |  | |  | |  | |  |  |  |  |  |  |  |
| NASA-TLX |  |  |  |  | |  | |  | |  |  |  |  |  |  |  |
| Total Score | 73.5 (58.25-85.75) | 65.5 (51.5-76.5) | 40 (26-52) | **0.0001** | | 0.2276 | | **0.001** | | **0.0001** | -1.2065 | -3.3026 | -3.8358 | -0.1760 | -0.4817 | -0.5595 |
| Mental Demand | 6 (5-10) | 9 (5.25-11.5) | 5 (2-10) | **0.0360** | | 0.2768 | | 0.2089 | | 0.7331 | 1.0875 | -1.2566 | -0.3410 | 0.1586 | -0.1833 | -0.0497 |
| Physical Demand | 7.5 (2.75-10) | 7.5 (5.25-10) | 8 (5-10) | 0.9895 | | 0.9239 | | 0.9201 | | 0.9523 | 0.0955 | -0.1003 | 0.0599 | 0.0139 | -0.0146 | 0.0087 |
| Temporal Demand | 20 (17.25-20) | 16.5 (13.5-20) | 10 (6-15) | **0.0002** | | 0.1024 | | **0.002** | | **0.0002** | -1.6331 | -3.0900 | -3.7034 | -0.2382 | -0.4507 | -0.5402 |
| Performance  (failure=20) | 5 (4.25-8.25) | 10 (5-12) | 14 (10-18) | **0.0016** | | 0.0447 | | 0.0335 | | **0.0011** | -2.0075 | -3.3383 | -4.4203 | -0.2928 | -0.4869 | -0.6448 |
| Effort | 19 (15.5-20) | 15 (10-17.5) | 10 (7-10) | **0.0002** | | 0.0129 | | 0.0145 | | **0.0003** | -2.4860 | -2.4438 | -3.6567 | -0.3626 | -0.3565 | -0.5334 |
| Frustration | 8.5 (3.5-14.75) | 7 (4.25-10) | 4 (2-7) | 0.0837 | | 0.5961 | | 0.0673 | | 0.0539 | -0.5299 | -1.8296 | -1.9276 | -0.0773 | -0.2669 | -0.2812 |

NASA-TLX: National Aeronautics and Space Administration-Task Load Index. OSATS: objective structured technical skills assessment.

Supplementary Table 3. Mental workload during ESS between experts and non-experts.

|  | **Non-experts**  **(n=36)** | **Experts**  **(n=11)** | **P value** | **Z score** | **Effect size (r)** |
| --- | --- | --- | --- | --- | --- |
| OSATS score | 51 (39.25-58) | 75 (72-78) | **<.0001** | 4.7514 | 0.6931 |
| Surgical experience in number of performed ESS cases | 30 (2-50) | 700 (300-1000) | **<.0001** | 4.8134 | 0.7021 |
|  |  |  |  |  |  |
| NASA-TLX |  |  |  |  |  |
| Total Score | 65.5 (53.75-79) | 32 (26-51) | **<.0001** | -4.1354 | -0.6032 |
| Mental Demand | 7 (5-10) | 6 (0-10) | 0.7031 | -0.3811 | -0.0556 |
| Physical Demand | 8 (5-10) | 6 (5-10) | 0.6841 | -0.40689 | -0.0594 |
| Temporal Demand | 18 (15-20) | 8 (2-10) | **<.0001** | -4.48429 | -0.6541 |
| Performance  (failure=20) | 13 (8-15) | 4 (0-6) | **<.0001** | -4.0808 | -0.6541 |
| Effort | 16.5 (15-20) | 10 (7-10) | **<.0001** | -3.9414 | -0.5749 |
| Frustration | 7 (4-11.75) | 4 (2-5) | **0.0461** | -1.9941 | -0.2909 |

NASA-TLX: National Aeronautics and Space Administration-Task Load Index. OSATS: objective structured technical skills assessment.

Supplementary Table 4. Comparison of registrars’ mental workload between 2^nd^ and final training.

|  | **2^nd^ training** | **Final training** | **P value** | **Z score** | **Effect size (r)** |
| --- | --- | --- | --- | --- | --- |
| OSATS score | 41 (35-46.75) | 60.5 (56.25-66.25) | **<.0001** | -2.8031 | -0.8864 |
|  |  |  |  |  |  |
| NASA-TLX |  |  |  |  |  |
| Total Score | 72 (63.5-77) | 66.5 (51-85.75) | 0.5382 | -0.6625 | -0.2095 |
| Mental Demand | 6 (2.75-10.25) | 12 (7-16) | **0.0023** | -2.5992 | -0.8219 |
| Physical Demand | 5.5 (4.75-11) | 10.5 (5.75-14.25) | **0.0213** | -2.2509 | -0.7118 |
| Temporal Demand | 20 (17.25-20) | 13 (11.25-20) | **0.0091** | -2.3664 | -0.7483 |
| Performance  (failure=20) | 15 (10-17.25) | 6.5 (3.75-12.25) | **0.0017** | **-2.5992** | **-0.8864** |
| Effort | 19 (16.25-20) | 16.5 (12.75-19.25) | **0.0346** | -2.1704 | -0.6863 |
| Frustration | 5.5 (3-10) | 7 (2.5-13.5) | 0.3625 | -1.0142 | -0.3207 |

NASA-TLX: National Aeronautics and Space Administration-Task Load Index. OSATS: objective structured technical skills assessment.
